# The Effect of Different Foot Orthosis Inverted Angles on Plantar Pressure in Children with Flexible Flatfeet

**DOI:** 10.1371/journal.pone.0159831

**Published:** 2016-07-26

**Authors:** Soo-kyung Bok, Hyunkeun Lee, Bong-ok Kim, Soyoung Ahn, Youngshin Song, Insik Park

**Affiliations:** 1 Department of Rehabilitation Medicine, Chungnam National University Hospital, Jung-gu, Daejeon, South Korea; 2 College of Nursing, Chungnam National University, Jung-gu, Daejeon, South Korea; 3 Korean Pedorthic Institute, Goyang, Gyeonggi-do, South Korea; West Virginia University School of Medicine, UNITED STATES

## Abstract

Although orthotic modification using the inverted technique is available for the treatment of flatfoot, empirical evidence for the biomechanical effects of inverted-angle foot orthoses (FOs) is lacking. The aim of this study was to evaluate the effects of different FO inversion angles on plantar pressure during gait in children with flatfoot. Twenty-one children with flexible flatfeet (mean age 9.9 years) were enrolled in this study. The plantar pressures were measured for the rearfoot; medial and lateral midfoot; and medial, central, and lateral forefoot as participants walked on a treadmill while wearing shoes only and shoes with the following 3 orthotic conditions: (i) orthosis with no inverted angle, (ii) orthosis with a 15° inverted angle, and (iii) orthosis with a 30° inverted angle. A one-way repeated measures analysis of variance (ANOVA) with the Bonferroni-adjusted post-hoc test was used to compare the mean values of each orthotic condition. Compared with the shoe only condition, the peak pressure decreased significantly under the medial forefoot and rearfoot with all FOs (p <0.05). However, no significant differences in the peak pressure under the medial forefoot and rearfoot were observed between the FOs. The peak pressure under the medial midfoot increased significantly with all FOs, and a maximal increase in the peak pressure was obtained with a 30° inverted angle orthosis. Furthermore, the contact area under the medial midfoot and rearfoot increased significantly with all FOs, compared with the shoe only condition (p <0.05). Again, no significant differences were observed between the FOs. For plantar pressure redistribution, a FO with a low inverted angle could be effective, accommodative, and convenient for children with flatfoot.

## Introduction

Flexible flatfoot is a condition in which the medial longitudinal arch (MLA) of the foot collapses during weight bearing and recovers after body weight removal [1,2]. Flatfoot, which may affect one or both feet, not only increases the load acting on the foot structure, but also interferes with normal foot function [3]. Over time, the mechanical overload resulting from the flattened MLA is transferred to proximal areas such as the knees, hips, and lower back [4].

Previously, many studies have examined plantar pressure in flatfoot subjects. Ledoux and Hillstrom [5] examined the effect of flatfoot on plantar vertical force distribution and reported significantly higher forces underneath the hallux relative to those measured in normal subjects. Queen et al. [6] conducted a comparative study to investigate the influences of flat and normal feet on force distribution during different athletic tasks. Their findings indicated that both the contact area and force in the medial midfoot were significantly higher in subjects with flatfoot. Ledoux and Hillstrom further noted that the peak force under the first submetatarsal area and hallux increased in the flatfoot when compared with a normally arched foot [5]. Pauk et al. [7] reported an increased pressure distribution and contact area under the medial midfoot in patients with flatfoot, compared with normal subjects. Furthermore, Song et al. [8] demonstrated medial deviation of the point at which the ground reaction force acts upon the foot in subjects with flat foot.

Foot orthoses (FOs) are frequently prescribed interventions for flexible flatfoot [9,10,11]. Such devices are designed to provide stability and realign the foot arch, and have yielded demonstrable success in the alleviation of patients’ symptoms [4,12,13,14]. Previous biomechanical studies have shown that orthotic insoles improve arch alignment, increase the duration of the stance phase of level walking, and reduce both the maximum foot pronation angle and tibial internal rotation [15,16,17].

Many types of orthotic styles, materials, and modifications have been designed to enhance the effects of FOs [18]. For example, Redmond et al. [19] examined the mechanical effects of customized (modified Root-type orthosis) and prefabricated FOs with longitudinal arch supports on pressure distribution within a group of subjects with flatfoot. The authors’ findings showed that both FOs shifted the load from the forefoot and rearfoot toward the midfoot area while increasing the midfoot contact area.

Foot pronation during the early stance phase of gait allows the foot to accommodate to ground surface irregularities and attenuate ground reaction forces. Pronation involves multiple joint movements at the rearfoot and midfoot and might thus influence more proximal segments leading to internal rotation of the lower limb and hip [20]. Clinicians have used foot orthoses in an attempt to control excessive pronation, and medial forces on the medial rearfoot and midfoot increase the action of the supination moment across the subtalar joint axis [21,22].

The Blake inverted technique was developed with the intention of improving the ability of a FO to control excessive foot pronation [23,24]. Specifically, different orthotic inversion angles can be prescribed, with greater angles indicated when greater pronatory control is desired. The inverted technique uses a 5:1 ratio to set the cast in an overcorrected position, with the goal of straightening the heel back to vertical based on a resting calcaneal stance position (RCSP) [23,24]. Therefore, a 5° correction of the rearfoot would require a device with a 25° inverted angle. Baitch et al. [25] compared the rearfoot mechanics of patients treated with both standard and inverted FOs and found that those with a 25° inverted angle more effectively controlled rearfoot pronation than did standard orthotic devices [25].

Despite clinical use, empirical evidence for the biomechanical effects of the inverted angle is lacking. In particular, a better understanding of the biomechanical effects on the foot will facilitate the prescription of FOs for the treatment of flatfoot and can provide clinicians with additional information for determining the inverted angles of FOs. Traditionally, children with flat feet have been treated with arch supports or corrective shoes to improve the arch, and several studies have evaluated the effectiveness of FOs in children with flatfoot. Jay et al. [26] reported significant improvements in the RCSP of children aged 20 months to 14 years with flexible flat foot. Previously, we studied the effects of inverted techniques FOs on radiological indicators in children with flatfoot and reported significant improvements in these indicators with inverted technique FOs in children with flatfoot [27]. However, little or no data concerning pressure/force, particulary in children with flatfoot, are available, and in our opinion, some children with prescribed high-degree inverted angle FOs complain of discomfort and thus exhibit poor compliance with FOs. Therefore, this study aimed to evaluate the effects of different rigid FO inverted angles on plantar pressure and force in children with severe flatfoot. It was hypothesized that the plantar pressure, contact area and force might differ with different orthosis inverted angles, and that an inverted angle of 15° or 30° might be most effective for children with a severe flat foot.

## Materials and Methods

### Participants

All participants were evaluated during >3 consecutive radiological studies and via RCSP measurements. The following radiological parameters were screened to evaluate the alignment of both feet: anteroposterior talocalcaneal angle (APTCA), lateral talocalcaneal angle (LTTCA), lateral talometatarsal angle (LTTMA), and calcaneal pitch (CP).

Flatfoot was defined as an angle of ≥4° in valgus in either foot during RCSP and one of the following abnormal radiological findings: APTCA >30°, LTTCA >45°, LTTMA >4°, or CP <20° [27,28,29]. We defined a severe flatfoot as an angle of ≥6° in valgus during RCSP. The following functional tests were performed to assess the ability to correct deformities: (a) the great toe extension test (Jack's test), and (b) the tip-toe standing test [30]. We recruited only symptomatic children aged 8–13 years of age with severe flexible flatfoot. The exclusion criteria were (i) a fixed foot deformity, (ii) reported previous intervention (e.g., orthoses or surgery), (iii) congenital and developmental foot disease, and (iv) neuromuscular or central nervous system disease. Patients with a history of overuse or traumatic injury to the lower limb in the past 6 months, bony surgery to the lower limb, or systemic endocrine, neurogenic, or musculoskeletal disorders were excluded.

A total of 21 participants with severe flatfoot were enrolled in this study, and 42 feet were evaluated for data collection. This study was approved by the Chungnam National University Hospital institutional review board (application number CNUH 2015-06-024-002), and written informed consent was obtained from all participants and legal parents. The patients’ age, sex, weight, and height were recorded (Table 1).

**Table 1:** Basic demographic characteristics of the participants (N = 21).

	Value
Age (years)	9.9 ± 1.6
Male: female	8: 13
Weight (Ibs)	85.3 ± 18.5
Height (cm)	136.5 ± 12.5
BMI	18.4 ± 2.4
RCSP	-8.6° ± 2.3°

Values are expressed as numbers or means ± standard deviations.

BMI, body mass index; RCSP, resting calcaneal stance position

### Foot orthoses

**Fig 1:**
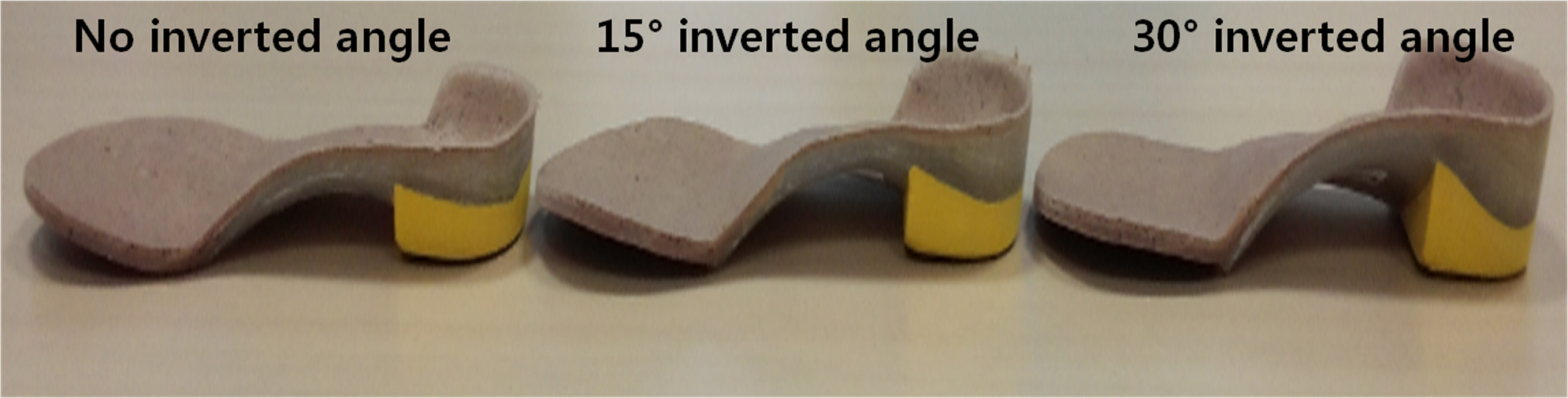
Medial view of the rigid foot orthoses. Left to right: (1) no inverted angle, (2) 15° inverted angle, (3) 30° inverted angle.

Participants underwent a standardized prone casting protocol to obtain neutral impression casts. The customized rigid FOs were designed in an “inverted” style and manufactured at a commercial orthosis laboratory (Biomechanics, Goyang, Korea) according to a strictly defined procedure and under the care of a single experienced technician. To accurately capture the negative foot impression, a plaster cast was handmade in a neutral, non-weight bearing position. The rigid orthotic shell was composed of 5-mm-thick polypropylene, and high-density ethylene vinyl acetate (EVA) was used for heel posting. The top cover was a mixture of low-density EVA and cork. This style of orthosis inverted the rearfoot and pronated the forefoot through the subtalar joint and longitudinal axis of the midtarsal joint. The FOs were manufactured with the following modifications: (i) no inverted angle, (ii) 15° inverted angle, and (iii) 30° inverted angle (Fig 1). The rigid FO with no inverted angle had a heel cup height of 10 mm, a contoured arch area to support the MLA, and a standard extrinsic rearfoot post.

### Biomechanical analysis

The Pedar-X in-shoe pressure system (Novel GmbH, Munich, Germany), which has been described in the literature [31,32,33], was used to measure plantar pressures and forces in both feet of each participant. The hardware includes flexible insoles comprising 84 capacitive sensors, a Pedar box (data logger) and battery pack (to be fastened on the subject’s waist), cables to attach the logger to the insoles, and a Bluetooth dongle. The proper size of Pedar insoles were used to accommodate the range of foot sizes in this study. Participants were asked to wear a pair of sport shoes with a firm heel counter that allowed easy placement of insole, rather than standard shoes. Each participant undertook 12 steps during each of 3 walking trials on a treadmill at a comfortable gait speed and cadence for each of the following 4 orthotic conditions: (i) shoe only, (ii) FO with no inverted angle, (iii) FO with a 15° inverted angle, and (iv) FO with a 30° inverted angle. To minimize the effects of acceleration and deceleration, only the middle 3 steps (whole stance phase) were used for data analysis. The 9 steps from the 3 trials were then averaged for each of the 4 orthotic conditions. The 4 orthotic conditions were tested in random order to minimize potential sequencing effects.

**Fig 2:**
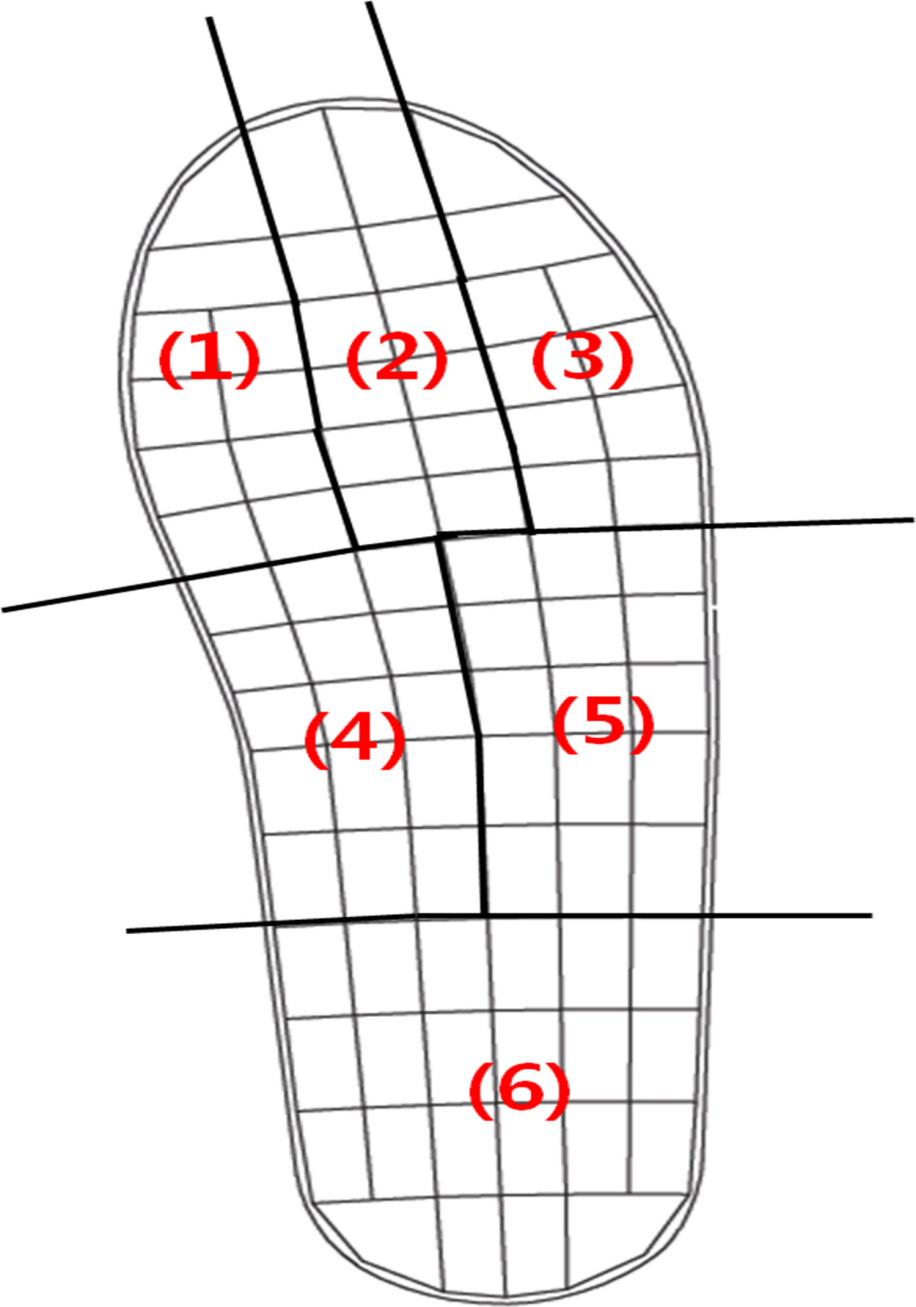
The 6 mask areas of the foot. (1) Medial forefoot, (2) central forefoot, (3) lateral forefoot, (4) medial midfoot, (5) lateral midfoot, (6) rearfoot.

Data were compared in 6 mask regions correspondi후35ng to anatomically relevant areas of the foot, namely the rearfoot, medial midfoot, lateral midfoot, medial forefoot (first toe and metatarsophalangeal region), central forefoot (second and third toes and metatarsophalangeal region), and lateral forefoot (fourth and fifth toes and metatarsophalangeal region) (Fig 2). Size percentage masks were applied to the rearfoot (proximal 27% of foot length), midfoot (middle 33% of foot length), and forefoot (distal 40% of foot length). According to the Pedar-X system output, the following 3 variables were calculated and analyzed for each mask: peak pressure (kPa), maximum force (N), and contact area (cm^2^). The maximum force was calculated as the summation of the peak pressure that was reached for each sensor at any time during the data collection in each region.

### Statistical analysis

All statistical analyses were performed using the SPSS software program version 18.0 (SPSS Inc., Chicago, IL, USA). The 9 steps (3 steps x 3 trials) for each condition were averaged and analyzed. A one-way repeated measures analysis of variance (ANOVA) with the Bonferroni-adjusted post-hoc test was used to compare means between each orthotic condition (data were normally distributed). Differences between orthotic conditions were considered statistically significant at a p-value <0.05.

## Results and Discussion

### Peak pressure

Compared with the shoe only condition, the peak pressure under the medial forefoot and rearfoot decreased significantly with all FOs (p<0.001), although no significant differences were observed between the FOs. The peak pressure under the medial midfoot increased significantly with all FOs (p<0.001), and a 30° inverted angle FO yielded a maximal increase in the peak pressure at the medial midfoot, compared with the other FOs (p <0.001).

Compared with the shoe only condition, the peak pressure under the central forefoot decreased significantly at a FO inverted angle of only 15° (p = 0.002). There were no differences in peak pressure between the orthotic conditions at any of the lateral forefoot or lateral midfoot masks (Table 2).

**Table 2:** Peak pressures at the 6 mask areas in each foot orthotic condition.

	Peak pressure(kPa)					
Masks	Shoe only	No inverted angle	Inverted angle 15°	Inverted angle 30°	F	p-value
Medial forefoot	411.24 (114.54)	309.95* (88.2)	304.83* (94.74)	317.62* (108.15)	24.82	<0.001
Central forefoot	232.13 (65.94)	188.95 (43.29)	176.24* (43.32)	189.55 (40.48)	7.5	0.005
Lateral forefoot	146.18 (51.72)	163.73 (69.02)	147.33 (44.88)	168.13 (41.45)	2.12	0.124
Medial midfoot	96.58 (55.46)	142.11* (58.43)	163.21*# (67.16)	184.16*#+ (81.43)	31.06	<0.001
Lateral midfoot	129.92 (66.56)	137.2 (59.85)	134.95 (55.51)	138.34 (55.22)	0.68	0.531
Rearfoot	269.39 (89.4)	136.33* (27.32)	140.63* (24.73)	143.94* (32.8)	59.57	<0.001

Values are expressed as mean (standard deviation)

*p<0.05 compared to the shoe only condition.

#p<0.05 compared to no inverted angle.

+p<0.05 compared to an inverted angle of 15°.

### Maximum force

Compared with the shoe only condition, the maximum force under the medial forefoot decreased at 15° (p = 0.002) and 30° (p<0.001) orthotic inverted angles. In contrast, the FO with no inverted angle did not significantly affect the maximum force.

The maximum force under the lateral forefoot increased significantly at a 30° inverted angle, compared with the shoe only condition (p<0.001). However, no significant changes were observed with other FOs.

Compared with the shoe only condition, the maximum force under the medial (all FOs, p<0.001) and lateral (no inverted angle, p = 0.003; 15° inverted angles, p = 0.008; 30° inverted angles, p = 0.007) midfoot increased with all FOs. The 30° inverted angle FO (p<0.001) yielded a significant increase in the maximum force under the medial midfoot when compared with the no inverted angle FO. In contrast, no such differences were observed between the FOs under the lateral midfoot. Compared with the shoe only (p = 0.012) and no inversion angle FO (p = 0.001) conditions, the maximum force under the rearfoot decreased significantly at a 30° FO inverted angle (Table 3).

**Table 3:** Maximal forces of the 6 mask areas in each foot orthotic condition.

	Maximal force(N)					
Masks	Shoe only	No inverted angle	Inverted angle 15°	Inverted angle 30°	F	p-value
Medial forefoot	186.24 (38.98)	166.11 (33.31)	158.39* (33.16)	152.15* (35.66)	10.41	<0.001
Central forefoot	168.12 (34.14)	172.18 (30.31)	168.32 (33.16)	171.29 (33.71)	1	0.381
Lateral forefoot	84.93 (13.33)	104.33 (23.53)	105.32 (24.14)	118.21* (27.12)	10.44	<0.001
Medial midfoot	72.18 (43.19)	118.13* (37.11)	129.99* (40.11)	137.14*# (35.32)	66.06	<0.001
Lateral midfoot	126.16 (44.41)	148.11* (42.31)	145.21* (41.93)	141.66* (45.2)	9.3	<0.001
Rearfoot	307.32 (65.46)	289.29 (64.13)	276.88 (75.31)	260.14*# (71.3)	8.5	0.002

Values are expressed as mean (standard deviation)

*p<0.05 compared to the shoe only condition.

#p<0.05 compared to no inverted angle.

### Contact area

Compared with the shoe only condition, the contact area under the medial midfoot and rearfoot increased significantly with all FOs (p <0.05), although there were no significant differences between the FOs. However, none of the FOs induced a significant change in the contact area under the lateral midfoot when compared with the shoe only condition. Furthermore, significant increases in the contact area under the lateral forefoot (p = 0.013) and decreases in the contact area under medial forefoot (p = 0.006) relative to the shoe only condition were observed only at a FO inverted angle of 30°. In other words, FOs with no and 15° inverted angles did not induce significant changes in the contact areas under the medial and lateral forefoot (Table 4).

**Table 4:** Contact areas of the 6 mask areas in each foot orthotic condition.

	Contact area(cm2)					
Masks	Shoe only	No inverted angle	Inverted angle 15°	Inverted angle 30°	F	p-value
Medial forefoot	16.99 (4.31)	16.64 (4.27)	14.91 (2.38)	14.33* (2.42)	8.77	<0.001
Central forefoot	23.89 (4.94)	25.44 (6.44)	24.79 (4.47)	25.31 (4.15)	3.2	0.075
Lateral forefoot	14.46 (2.46)	16.42 (3.59)	16.56 (3.8)	17.69* (4.85)	6.24	0.004
Medial midfoot	16.31 (4.43)	19.98* (3.51)	19.73* (3.55)	20.94* (4.91)	9.23	0.003
Lateral midfoot	24.58 (4.44)	25.19 (3.67)	25.53 (4.24)	25.09 (3.31)	0.98	0.373
Rearfoot	35.01 (4.51)	39.89* (6.94)	38.27* (6.56)	39.31* (7.12)	10.63	<0.001

Values are expressed as mean (standard deviation)

*p<0.05 compared to the shoe only condition.

### Discussion

Many types of orthotic styles, materials, and modifications have been designed to enhance the effects of foot orthoses [18]. For example, the medial heel skive technique was developed with the intention of improving the ability of a foot orthosis to control excessive foot pronation [34]. Different medial heel skive depths can be prescribed, and greater depths are indicated when greater pronatory control is desired [34]. Bonanno et al. [21] evaluated the effects of different medial heel skive depths on plantar pressures and reported that a medial heel skive of 4 or 6 mm increased the peak pressure under the medial rearfoot. However, there is little data regarding the effects of FO inverted angles on biomechanical factors such as plantar pressure. Accordingly, this study evaluated the effects of different FO inverted angles on plantar pressure in children with severe flexible flatfoot.

The findings of this study suggest that significant decreases in medial forefoot and rearfoot peak pressure could be achieved in children with severe flatfeet using FOs at all tested inverted angles, although the effects of these changes on kinematic motion in the rearfoot remain unknown. However, Mueller et al. [35] reported that a reduction in peak plantar pressure on the forefoot during walking contributed to the prevention of calluses, foot deformities, reduced plantar tissue thickness, and limited joint mobility. We expected that an FO with no inverted angle would have a minor effect on pressure distribution. Although an increase in peak pressure at the medial midfoot was observed with a greater FO inverted angle, unfortunately we did not observe a corresponding decrease in peak pressure at the medial forefoot and rearfoot with a greater FO inverted angle. We considered that the effect of the FO with no inverted angle on peak pressure redistribution might have been due to the medial longitudinal arch support provided by the contoured arch area. However, the long-term effects of a FO with no inverted angle remain uncertain.

The peak pressure and contact area under the medial midfoot increased significantly with all FOs; however, similar changes were not observed under the lateral midfoot. We found that the use of FOs with or without inverted angles shifted the load from the forefoot and rearfoot toward the medial midfoot, compared with the shoe only condition. This shift in load toward the medial midfoot was associated with a concomitant increase in the medial midfoot contact area, which minimized changes in pressure in this region. These results correspond to the findings of Redmond et al. [19], who also observed a shift in load toward the midfoot when using contoured FOs (prefabricated or customized, modified Root-type) and findings of McCormick et al. [36] and Bonanno et al. [37].

In this study, only a FO with a 30° inverted angle yielded significant increases in the maximum force and contact area at the lateral forefoot. The increased maximum force and contact area at the lateral forefoot might cause skin irritation and discomfort on the little toe, although this is uncertain. All FOs yielded significant increases in the maximum force under the medial midfoot when compared with the shoe only condition. Simultaneously, the maximum force under the lateral midfoot increased significantly with all FOs. However, with all FOs, the maximal force at the medial midfoot increased at much greater rates (65–92%) than did the increase at lateral midfoot (11–16%), suggesting that the use of a FO will increase the midfoot supination force, thus contributing to the control of excessive pronation.

The study had the following limitations. First, we did not subdivide the mask under the rearfoot into lateral and medial areas. Accordingly, we could not evaluate whether the FO inverted angle medially shifted the force acting on the medial plantar heel. Such an increase in medial force is thought to accompany a concomitant decrease in the force to the lateral plantar heel. Second, standardized footwear was not used to minimize the potential influence of footwear on the results. It is uncertain how the results of the study would have differed if the orthoses had been tested in more supportive footwear. Third, although we did not detect a major difference between FOs, this is more likely to be related to an underpowered statistical design rather than a true non-difference. It is unknown how much of a decreased pressure/force is necessary for a beneficial effect, or an increased pressure/force is deleterious. Fourth, despite the demonstrated reliability and validity of the Pedar-X system, it could only measure forces acting perpendicular to the insole [31,32,33]; however, it is likely that pressures under the foot are more complex. Fifth, the sample size was relatively small. Specifically, we analyzed pressure data under only 42 feet of 21 children, leading to concerns regarding problems associated with the statistical analysis of paired data collected from 2 feet or from 1 person during biomechanical analyses. Sixth, because the present study was only conducted for a short time period, the results may not be generalizable without further studies of the long-term effects.

## Conclusion

When using the inverted technique, rigid FOs yielded significant decreases in the peak pressure under the hallux and heel regardless of the inverted angle. However, we did not observe an association between an increased FO inverted angle and a decrease in peak pressure under the hallux and heel. Regarding plantar pressure redistribution, a lower FO inverted angle could be effective, accommodative, and convenient for children with flatfoot.
